# Chromosome Abnormalities Related to Reproductive and Sexual Development Disorders: A 5-Year Retrospective Study

**DOI:** 10.1155/2021/8893467

**Published:** 2021-05-05

**Authors:** Sara Benchikh, Amale Bousfiha, Lunda Razoki, Jamila Aboulfaraj, Latifa Zarouf, Chadli Elbakay, Lala Laila Rifai, Adil El Hamouchi, Sanaa Nassereddine

**Affiliations:** ^1^Laboratory of Cytogenetics, Pasteur Institute of Morocco, Casablanca, Morocco; ^2^Laboratory of Physiopathology and Molecular Genetics, Faculty of Sciences Ben M'Sik, Hassan II University, Casablanca, Morocco

## Abstract

Chromosomal abnormalities are the main genetic risk factor associated with reproductive and sexual development disorders (DSD). The goal of this study is to retrospectively evaluate the frequency of chromosomal aberrations in Moroccan subjects with problems of procreation or sexual ambiguity. A total of 1005 individuals, including 170 infertile couples, underwent cytogenetic analysis in the Cytogenetic Laboratory of the Pasteur Institute of Morocco. Heparinized blood samples were processed according to the standard karyotype method. A total (81.5%) of the patients studied had a normal karyotype, while the remaining (18.5%) patients had an abnormal karyotype. Female patients had more chromosomal abnormalities (52%) than male patients (48%). These chromosomal aberrations included 154 cases (83%) of sex chromosomal abnormalities, the most common being Turner's syndrome and Klinefelter's syndrome, and 31 cases (17%) had autosomal aberrations, especially chromosome 9 reversal (inv(9)(p12;q13)). The present data shows that among 170 couples, 10.6% had chromosomal abnormalities mainly involved in the occurrence of recurrent miscarriages. Genotype-phenotype correlations could not be made, and therefore, studies using more resolutive molecular biology techniques would be desirable.

## 1. Introduction

Disorders of sex development are known for being an abnormal development of the internal and external genital organs [1].

As for disorders of reproduction, they are defined as pathologies that can affect reproductive function in humans and therefore cause conception failure [2]. The alteration of human reproductive function can be due to organic, congenital, functional, accidental, or genetic (such as chromosomal abnormalities) disorders.

Chromosomal abnormalities are more common in groups of patients with sexual ambiguity or unexplained infertility, and their chance of conceiving varies because sometimes sterility is inevitably the direct consequence of the revealed anomaly.

The abnormalities could be specific to sex chromosomes such as Turner syndrome, Klinefelter syndrome, sex reversal (also called De la Chapelle syndrome for XX males), Jacob syndrome, triple X syndrome, and mixed gonadal dysgenesis. Structural rearrangements such as microdeletions of chromosome Y involving AZF factor which is crucial for spermatogenesis, Y isochromosome: on the one hand, Yp isochromosome is implicated in male infertility since the AZF region is lost and the region of male determinism SRY is retained; on the other hand, Yq isochromosome is associated with female determinism with the presence of the AZF region [3]. Translocations between the Y chromosome and an X can lead to abnormal phenotypes causing sexual ambiguity or infertility [4]. Ring Y chromosome is characterized by a wide spectrum of phenotypes such as anomalies in genital organs, hypogonadism, oligospermia, or azoospermia [5]. Xq isochromosome often encountered in Turner's syndrome is characterized by amenorrhea, ovaries that function normally but are atypical (fibrous strips). Deletion of Xp can lead to gonadal dysgenesis, infertility, or amenorrhea depending on the breakpoint [6, 7]. A translocation between X and an autosome, when occurred in the POF1 and POF2 locus, results in ovarian disorders [8]. For autosomal chromosomes, structural abnormalities such as reciprocal translocations and Robertsonian translocations present an increased risk of miscarriages and male infertility due to the generation of imbalanced gametes [9, 10]. Regarding female infertility, these abnormalities lead to the production of unbalanced oocytes carrying structural abnormalities which can be the cause of failed fertilization or implantation and spontaneous miscarriages [11].

In this report, we focused mainly on determining the frequencies and types of chromosomal abnormalities, detected by karyotype, which are the cause of reproductive and sexual development disorders in Morocco.

## 2. Materials and Methods

### 2.1. Patients

A retrospective study was conducted on 1005 patients with reproductive disorders or DSD in the cytogenetic department of Pasteur Institute of Morocco over a period of 5 years (from January 2014 to December 2018). Among these 1005 individuals, 340 constitute 170 infertile couples. These patients were referred with various clinical information mostly miscarriages, sterility, Turner suspicion, amenorrhea, and azoospermia (Table 1).

### 2.2. Karyotype Analysis

For the diagnosis of patients, a cytogenetic analysis (karyotype) was carried in order to determine the types and frequencies of chromosomal aberrations implicated in these pathologies. After collecting peripheral blood in heparinized tubes, lymphocyte culture was achieved in Roswell Park Memorial Institute medium (RPMI-1640) with phytohemagglutinin. The samples were incubated at 37°C for 72 hours. By the end of cell culture, colchicine is added. We incubate again our samples for 1 hour at 37°C; then, we centrifuge. After centrifugation, a hypotonic solution is added (0.2 M MgCl_2_·6H_2_O). Subsequently, the fixation is performed with Carnoy's fixative. We incubate at +4°C for 25 min; then, we centrifuge. This step is carried out several times until a clear pellet is obtained on which we add more fixative. The fixed cell suspension will be spread on a slide, dried, and then incubated at 37°C. The thermal denaturation process for R bands (also called RHG) consists of treating the slides in a bain-marie at 86°C in the presence of Earle's solution, and it is stopped (the denaturation) after rinsing the slides with water. The slides will be dipped in Giemsa for staining. Finally, the chromosomal preparations are viewed in a microscope coupled to a computer.

In all the cases studied, at least 20 metaphases were selected and analyzed. In the case of mosaicism, 50 cells were analyzed. Patients' data were collected on an Excel file and were processed by Power BI software which made it possible to visualize them graphically. Note that all the chromosomal rearrangements and variants have been taken into account and chromosome nomenclature was done according to ISCN 2016.

### 2.3. Statistical Analysis

A chi-square test was carried out to test the difference between our results and other results reported in different studies using SPSS version 22.0. The confidence interval (CI) was set at 95%, and the results were considered statistically significant at *p* < 0.05.

## 3. Results

Cytogenetic analysis revealed that, among 1005 patients, only 185 have chromosomal abnormalities related to reproductive disorders and DSD. These various chromosomal abnormalities involve both sex chromosomes and autosomes. The age range of the patients studied varies from newborn to 60 years old. In this chapter, we will proceed on the one hand to a global analysis of the patient's results and on the other hand to a specific analysis of couples with infertility.

### 3.1. Global Analysis

**Figure 1:**
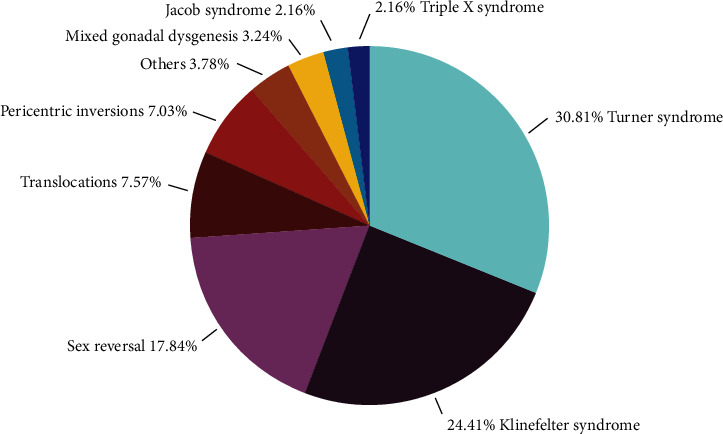
Different anomalies detected in patients. Others = deletion, addition, satellite chromosome, chimerism, marker chromosome.

The first analysis using Power BI (Figure 1) showed a predominance of Turner's syndrome (30.81%), followed by Klinefelter's syndrome (25.41%), sex reversal (17.84%), translocations (7.57%), pericentric inversions (7.03%), mixed gonadal dysgenesis (3.24%), Jacob's syndrome and triple X syndrome (2.16% each), and others (3.78%) which combine deletions, a satellite chromosome, an addition, a chimerism, a derivative of Y, and a marker chromosome.

**Figure 2:**
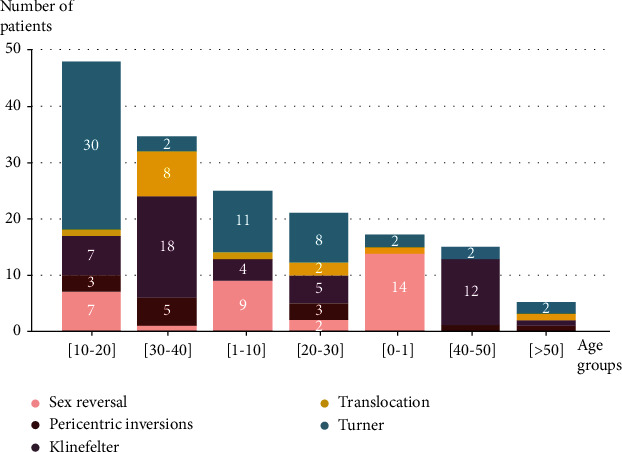
Distribution of the main chromosomal rearrangements found according to age.

A distribution of the main chromosomal abnormalities observed according to the age groups of the patients is shown in Figure 2. The histogram shows that the age group between [10–20] is the one with the highest number of diagnosed cases (51/185), followed by the age group [21–31] (36/185). Turner's syndrome is diagnosed mainly between [10–20] (30/57). Klinefelter syndrome, translocations, and pericentric inversions are more concentrated in the 30 to 50 age range group. Sex reversal and mixed gonadal dysgenesis are diagnosed especially during childhood from newborn to 10 years old (Figure 2).

Chromosomal aberrations were then classified into gonosomal abnormalities and autosomal abnormalities. The karyotype details of the different sex chromosome abnormalities have been listed in Table 2 with a highlight of the different variants detected in our Moroccan patients. These variants are either numerical aberrations or structural aberrations or both.

Autosomal abnormalities are summarized in Table 3. This table shows mainly structural abnormalities such as reciprocal translocations, Robertsonian translocations, pericentric inversions, an addition, and a deletion.

**Figure 3:**
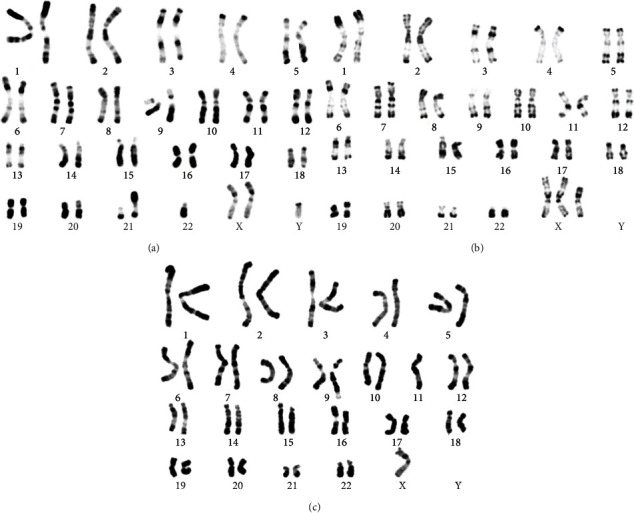
(a) Blood karyotype in RHG tape showing a 46,XXY,t(21;22)(q10; q10) formula. (b) Blood karyotype in RHG tape showing a 47,X,der(X)t(X;X)(q12; q11.3), der(X)t(X;X)(q11;q11.3) formula. (c) Blood karyotype in RHG tape showing a 45,X,t(Y;9) (p11;q34) formula.

The blood karyotyping performed revealed some rare karyotypes characterizing different pathologies of which 2 included both autosomal and gonosomal abnormalities and one only gonosomal aberrations. These results are presented in Figure 3: a variant of Klinefelter's syndrome with a translocation between 2 acrocentric chromosomes (Figure 3(a)), a variant of Turner's syndrome with a complex karyotype of a cell line included in a Turner's syndrome mosaicism (Figure 3(b)), and a translocation between an autosomal chromosome and a sex chromosome (Figure 3(c)).

### 3.2. Couple Analysis

In this study, 170 couples have come to the Cytogenetic Laboratory of Pasteur Institute of Morocco during the past 5 years. According to the reason for consultation, 134 couples came for redundant miscarriages, 24 for sterility, 10 for infertility, and 2 for failure of in vitro fertilization. Out of 170 couples, 18 couples had different types of chromosomal abnormalities (Table 4). Structural aberrations were observed in 15 couples, and numerical aberrations were observed in 3 couples. The partner affected was a woman in 7/18 couples (38.9%) and a man in 11/18 couples (61.1%). The majority of the aberrations found were involved in miscarriages in 16/18 couples (88.8%). Translocations counted for 4.7% of all the aberrations detected and pericentric reversal of chromosome 9 were detected in 3.5% of all couples.

## 4. Discussion

In our study, a retrospective analysis was performed on the karyotype results of 1005 individuals with infertility or sexual development disorders. It should be noted that the karyotype technique was able to detect 185 chromosomal abnormalities. Among other patients, it is necessary to use other more resolving and efficient techniques to identify indistinguishable abnormalities such as microdeletions or mutations in case they exist. The distribution of the main anomalies by age group reveals that the highest rate is between 10 and 20 years, especially for Turner's syndrome linked to delayed puberty and growth. However, between the ages of 30 and 50 where Klinefelter's syndrome predominates, they are mainly related to conception failure since it is at this age range that these people are more willing to conceive and therefore consult for infertility issues.

Sexual development disorders including sexual ambiguity and hermaphroditism are especially observed at a very young age from newborns to 1 year old. In this particular age group, complicated cases require more in-depth evaluations and consultation with the parents for the orientation of the treatment, which can be surgical. For example, in the case of sex conversion to male, suppression of the female canal structures will be carried out, hence the interest of diagnosis from a young age.

### 4.1. Gonosomal Abnormality Description

Cytogenetic analysis of our patients revealed for Klinefelter syndrome the variant 48,XXYY,22S+ which is very rare and has an incidence of 1/45,000 males [12]. Phenotypically, it has been described as being similar to Klinefelter but with additional abnormalities such as facial dysmorphies and respiratory diseases [13].

For Turner's syndrome, karyotype 45,X was associated in one case with sexual ambiguity; a study suggests that 10% of individuals with this karyotype have a Y chromosome hidden in other tissues, which explains this phenotype [14].

Mosaic 45,X/46,XX describes subjects with a normal phenotype rather than a classic Turner phenotype. Among them, the frequency of menstruation reaches 20%, but among pure Turner patients, it does not exceed 3% [15].

Patients with the karyotypes 45,X/46,X,mar+ and 45,X/46,X,mar+/47,X,+mar x2 show clinical sign characteristic of Turner (primary amenorrhea, short stature, growth retardation,…). And therefore according to the clinical diagnosis and the cytogenetic result, they could be classified in the category of Turner's syndrome. However, the application of FISH technique and PCR remains important to determine the nature of these markers and to know whether it is a fragment of the X or Y chromosome. This is useful in order to prevent the development of gonadal cancer in these women if the marker is a Y chromosome [16]. 45,X/47,XXX is a rather rare karyotype in which the patients do not present observable Turner's stigmata. However, ovarian failure is noted [17].

The long arm isochromosome of chromosome X with or without mosaic 46,X,i(Xq) or 45,X/46,X,i(Xq) remains the most common of Turner's variants found in this study; they were referred for amenorrhea and suspicion of Turner. One of the most observed characteristics is a reduced size explained by haploinsufficiency of the SHOX gene [18].

The derivative of chromosome X in mosaic 45,X/46,X,der(X) or the duplication of the X in mosaic 45,X/46,X,dup(X) leads to the usual clinical features of Turner's syndrome. The complex case of the X derivative mosaic with the 3 cell lines has a dominant cell population 45,X.

The mosaic 45,X/46,X,r(X) generates typical Turner symptoms with a high frequency of brain abnormalities and learning disabilities in addition to syndactyly and features of Kabuki syndrome in some cases [19].

The karyotype 46,X,del(X)(p21 → pter) that we have identified include the region containing the SHOX gene, and its deletion is linked to growth retardation. Genes being located on the short arm are also concerned since they are necessary for normal development of the cardiovascular system and therefore their deletion leads to cardiovascular pathologies [20].

Triple X syndrome 47,XXX is characterized by large size patients, hypotonia, and clinodactyly due to an overexpression of genes. These women have an increased risk of developing early ovarian failure [32].

Jacob's syndrome 47,XYY is manifested by infertility in all of the cases received, except for one case of mosaicism found in a patient with facial dysmorphism. The prevalence of this syndrome is similar to Klinefelter, but it is rarely identified and may not receive medical attention since it is not associated with clinical signs of infertility [33].

According to literature, male sex reversal syndrome's patients without sexual ambiguity (5/12 in our case) are SRY+ and have characters similar to Klinefelter syndrome, and those with sexual ambiguity (7/12 in our case) are SRY- and have hypospadias or insufficient degrees of virilization in the external genitalia. Reports suggest that a duplication/triplication of S0X9 and a duplication of the S0X3 gene can lead to the phenotype of the male SRY- [34].

Female sex reversal syndrome patients have been diagnosed with primary amenorrhea, sexual ambiguity, facial dysmorphism, or psychomotor retardation. Although the SRY gene is essential for the formation of the male phenotype, in these patients, it can be either a Y chromosome having lost SRY or a Y chromosome carrying a mutated SRY causing gonadal dysgenesis [5].

Patients with mixed gonadal dysgenesis have a broad phenotypic spectrum including normal women or women affected with Turner's syndrome, men with hypospadias, and male or female pseudohermaphrodism. Of the 6 reported cases of MGD, 5 were presented for sexual development disorder and one for psychomotor retardation. The karyotype shows a presence of the line 45,X which is frequently associated with rearrangements of the Y chromosome such as the isodicentric Y.

The karyotype 45,X/47,X,+mar1,+mar2 is a mixed gonadal dysgenesis deduced from the literature and clinical diagnosis which indicates sexual ambiguity. The following case 46,X,der(Y)add(?;Y) with a Y derivative and the presence of chromosome material of unknown origin on the Y chromosome had also sexual ambiguity. Both cases necessitate the search for the SRY region by PCR or FISH to determine the sex of the individual and to be able to perform sex corrective surgery if necessary [35].

The translocation between Y and 9 is observed in an azoospermic man. The breakpoint involved is assumed to be in the euchromatic distal region Yq11 of the azoospermia factor (AZF) locus near Yp11 [36].

The case of chimerism 46,XY/46,XX was manifested by an ambiguous phenotype. To explain the mechanism of chimerism, hypotheses have been put forward: first, double fertilization of an egg and its second polar body, and next, a fusion of two eggs fertilized separately [37].

Our results on gonosomal abnormalities were compared to other similar studies performed in Oman by Al-Alawi et al. (2016) and in Casablanca, Morocco, by Elkarhat et al. (2019). In our study, the frequency of Klinefelter's syndrome (25.4%) is higher than both frequencies reported by Al-Alawi et al. (24%) and Elkarhat et al. (1.41%). The frequency of Turner's syndrome (30.8%) is higher than that found by Elkarhat et al. (9.39%) and lower than the result of Al-Alawi et al. (38%). For sex reversal, our rate (17.8%) is higher in comparison to Elkarhat et al. (0.63%) but it remains lower than the frequency found by Al-Alawi et al. (19.4%). The frequencies of sex chromosome anomalies we obtained were comparable (*p* > 0.05) to frequencies reported by Al-Alawi et al. and statistically significant (*p* < 0.05) with that observed in Elkarhat et al. study.

These variations are due to the selection criteria of the patients recruited. Indeed, Elkarhat et al. have targeted a population with the clinical spectrum of ambiguous genitalia, amenorrhea, and Turner phenotype only which explains the low percentage of Klinefelter's syndrome and sex reversal; the low percentage of Turner's syndrome detected is due to large sample size. On the other hand, the study by Al-Alawi et al. primarily targets patients with suspected sex chromosome abnormalities only and has a study spread between 1994 and 2014 which explains the high rates of these abnormalities. The frequencies in Table 5 are recalculated relatively to the total number of abnormities found in each study.

### 4.2. Autosomal Abnormality Description

The reciprocal translocations found include several chromosomes which are 1, 2, 3, 5, 7, 8, 9, 10, 11, 13, 18, and 19. Most of them have already been reported in the literature [21–23, 40]. These patients with reciprocal translocations have a considerably increased risk of infertility and miscarriages mainly due to a generation of unbalanced gametes.

Robertsonian translocations involve the acrocentric chromosomes (13, 14, 15, and 21) and are found in patients with infertility. For example, rearrangements involving chromosomes 13 and 14 increase the risk of early pregnancy loss [24].

The inversions found are all of the pericentric types and involve chromosomes 2, 8, 9, and 12. The correlation between inversions and infertility will be discussed in the section of couple infertility. The variant 9phqh has no pathological significance.

This karyotype 46,XY,add(11)(q25) resulted in an ambiguous phenotype. The relationship between this rearrangement and the resulting phenotype has not been elucidated in the literature and therefore requires further research.

Regarding deletions, 18q deletion has occurred in a young girl with amenorrhea. Deletion of the long arm of chromosome 18 has an incidence of 25/1,000,000 among newborns. Del(18q) syndrome is frequently characterized by short stature, microcephaly, lack of myelination of the brain, and very little by primary hypogonadism and amenorrhea [25].

The other del(8) deletion mosaic with a normal 46,XY cell line in a man is already cited in the literature as being involved in spontaneous abortions [26].

The carrier of the supernumerary chromosomal marker presents a sexual ambiguity. Generally, these are de novo events which involve acrocentric chromosomes and do not present any identifiable phenotype in 2 thirds of the cases. The rest have clinical features, ranging from subfertility to birth defects or intellectual disability [10]. Their implication in sexual development disorders remains a question mark.

The consequence of the satellite chromosome present on chromosome 22 results in a miscarriage. Regarding the incrimination of the satellite chromosome in infertility reports diverge. Some have reported the presence of this variant in various clinical conditions such as reproductive failure or spontaneous abortions or even psychiatric disorders [27].

Our results regarding autosomal abnormalities involved in reproductive disorders were compared with other studies done in Portugal, Ukraine, and Egypt. In our study, 980 individuals had known sex (men or women) and 179 abnormalities and therefore were selected for this comparison. The rest were of unknown sex.

In Table 6, the number of patients received is indicated (*N*) as well as the number of anomalies discovered in each sex or both. The highest rates of translocations (60.49%) and inversions (18.51%) are detected among women and men in Ukraine while the lowest rates of translocations (7.82%) are detected in Morocco and inversions (5.88%) in Egypt. Deletions are rarely found during these studies with a rate of only 1.11% in Morocco and 0.42% in Portugal. Statistical analysis regarding genders showed that the results reported in Ukraine were in accordance (*p* > 0.05) with ours, but those reported in both Egypt and Portugal showed significant statistical difference (*p* < 0.05).

It is noted that the study carried out in Ukraine is done over a period of 5 years and mainly targeted infertile patients as in our report but the only difference is the number of patients recruited in the study made in Ukraine is 3.5 times superior to ours. This shows that these abnormalities are implicated in infertility.

In contrast, the studies in Egypt and Portugal have been done on a broad spectrum of patients who constitute couples with chromosomal abnormalities that will undergo assisted reproductive technology. These couples had mainly sex chromosome abnormalities.

### 4.3. Couple Infertility Analysis

In our study on couples, we found that (16/18) chromosomal aberrations are involved in miscarriages and therefore we will focus on the relationship between miscarriages and chromosomal rearrangements. These miscarriages are caused by fetal aneuploidies due to the parent with a balanced translocation. The risk of miscarriages with inversion is not common because it causes neither gain nor loss of genetic material, but in our case as well as other reported cases, the pericentric inversion especially that of chromosome 9 participates in spontaneous abortion and infertility in couples, and according to Vijay et al., the most common one is inv(9)(p12q13) [30]. There are contradictory views on its clinical impact; some believe that it is a normal variant, while others associate it with several pathologies such as infertility and obstetric history. The relationship between this variant and infertility is still unclear and requires further study to understand it.

Most studies have reported the predominance of chromosomal abnormalities in women, unlike our survey which showed that men had more abnormalities than women. The variation in the number of miscarriages compared to age does not provide any important information since young women who are less than 25 years old, with several miscarriages, have been identified, and therefore, the hypothesis of the association of advanced maternal age with more miscarriages is rejected.

Different published articles have reported the different anomalies causing infertility in couples, and their frequency varies between 3.5% and 11% according to Table 7. In this table, our results were comparable (*p* > 0.05) to results found in Morocco (11%), India (9.8%), Brazil (7.2%), Egypt (9%), and Tunis (6.9%) but were statistically significant (*p* < 0.05) compared to studies conducted in Iran (5.9%) and the United Kingdom (3.5%). These variations can be caused by sample size, geographic location, cultural differences, ethnicity, or criteria for case selection.

## 5. Conclusion

Following the cytogenetic analysis carried out in this work, a variety of chromosomal aberrations involved in reproductive disorders and DSD have been detected in the Moroccan population. Among all the anomalies, aneuploidies are the most dominant (51%). Gonosomal abnormalities are more frequent with a preponderance of Turner (30.81%) followed by Klinefelter syndrome (25.41%). Men have more translocations and gonadal dysgenesis and women more pericentric inversions and sex reversal. The age group between 10 and 20 includes the highest number of pathological cases diagnosed (51/185). In the infertile couple, men are more affected by chromosome rearrangements, and among these rearrangements, the most common is the pericentric reversal of chromosome 9 involved in the occurrence of miscarriages.

In order to explore all the chromosomal anomalies, conventional cytogenetic remains limited. It is therefore necessary to use the FISH technique which is more resolving. This technique, recently implemented in the Cytogenetic Laboratory of the Institute Pasteur, remains a promising prospect in so far as it allows to screen patients with reproductive or sexual development disorders by detecting, for example, microdeletions of the AZF region and alterations at the *SRY* locus.

The process of identifying the etiology of genetic pathologies related to infertility using these techniques helps predict the patient's phenotype. However, an association between a genetic cause and the consequent phenotype constitutes a real challenge for clinicians who find themselves faced with a probability of misdiagnosis.

As for infertility treatments, despite the scientific progress made over the past three decades which have helped millions of people to resolve their fertility problems (through drug treatments or assisted reproduction techniques such as in vitro fertilization), certain genetic causes linked to reproductive disorders/DSD remain in some cases intractable.

## Figures and Tables

**Table 1:** Clinical information of the 1005 patients selected for this study.

	Number of patients	%
*Males* (*n* = 471)		
Sterility	157	15.6
Miscarriages	151	15
Azoospermia	43	4.3
Infertility	32	3.2
Suspicion of Klinefelter	23	2.3
Hypogonadism	22	2.2
Sperm abnormalities	18	1.7
Genital malformation	10	1
Others^1^	16	1.6
*Females* (n =467)		
Miscarriages	179	17.8
Turner's suspicion	89	8.8
Primary/secondary amenorrhea	79	7.8
Sterility	57	5.7
Infertility	16	1.6
Growth retardation	15	1.5
Hormonal disorder	9	0.8
Hypogonadism	3	0.3
Others^2^	21	2.1
Disorder of sex development	67	6.7
Total	1005	100

Others^1^ = pubertal delay, in vitro fertilization (IVF), failure to thrive, facial dysmorphism. Others^2^ = reduced form uterus, facial dysmorphia, inguinal hernia, ovarian dysgenesis, psychomotor retardation.

**Table 2:** Karyotype characteristic of gonosomal anomalies.

Types of disorder	Cytogenetic grade	Karyotype	Cases
Klinefelter syndrome *n* = 47	Pure Klinefelter	47,XXY	45
	Aneuploidies	46,XXY,t(21;22)(q10;q10)	1
		48,XXYY,22S+	1

Turner syndrome *n* = 57	Pure Turner	45,X	23
	Turner's mosaic with number aberration	45,X/46,X,+mar	7
		45,X/46,XX	5
		45,X/46,X,+mar/47,X,+mar x2	1
		45,X/47,XXX	1
	Turner's mosaic with structural anomalies	45,X/46,X,i(Xq)	8
		45,X/46,X,der(X)	2
		45,X/46,X,idic(X)(p23)	1
		45,X/46,X,del(Xp)	2
		45,X/46,X,dup(X)(q13q28)	1
		45,X/46,X,der(X)t(X;X)(q12q11.3)/47,X,der(X)	1
		t(XX)(q12q11.3),der(X)t(XX)(q11q11.3)	
		45,X/46,X,r(X)	1
		45,X/46X,dic(X)(pter → q?)/der(X)	1
	Structural abnormalities of chromosome X	46,X,i(X)(q10)	2
		46,X,del(X)(p21 → pter)	1

Triple X syndrome *n* = 4		47,XXX	2
		48,XXX,+21	1
		47,XXX,t(13;21)(q12;q22)	1

Jacob's syndrome *n* = 4		47,XYY	2
		46,XY/47,XYY	2

Male sex reversal *n* = 12		46,XX	11
		47,XX,+21	1

Female sex reversal *n* = 21		46,XY	20
		46,XY,del(6)(q25)	1

Mixed gonadal dysgenesis *n* = 6		45,X/46,XY	2
		45,X/46,X,idic(Y)(p11.3)	1
		45,X/46,X,idic(Y)(q12)	1
		45,X/46,X,idic(Y)(p11)/47,XYY	1
		45,X/47,X,+mar1,+mar2	1

Y structural abnormalities *n* = 2	Derivative	46,X,der(Y)add(?;Y)	1
	Translocation	45,X,t(Y;9)(p11;q34)	1

Chimerism		46,XY/46,XX	1

**Table 3:** Karyotype characteristic of autosomal anomalies.

Translocations *n* = 13	Reciprocal	*Male karyotypes*	
		46,XY,t(1;9)(q41;p23)	1
		46,XY,t(8;8)(p22;q23)	1
		46,XY,t(13;19)(q33;q11)	1
		46,XY,t(3;18)(q28;q22)	1
		46,XY,t(2;5)(p23;q35)	1
		*Female karyotypes*	
		46,XX,t(1;7)(p16;q11)	1
		46,XX,t(1;19)(p32;q13)	1
		46,XX,t(5;10)(p15;q16)	1
		46,XX,t(2;11)(q32;q21)	1

Translocations *n* = 13	Robertsonian	*Male karyotypes*	
		45,XY,rob(14;21)(q10;q10)	1
		46,XY,rob(13;13)(q10;q10),+13	1
		*Female karyotypes*	
		45,XX,rob(21;21)(p10;p10)	1
		45,XX,rob(13;15)(q10;q10)	1

Pericentric inversions *n* = 13		*Male karyotypes*	
		46,XY,inv(9)(p12q13)	5
		46,XY,inv(2)(p11;q13)	1
		*Female karyotypes*	
		46,XX,inv(9)(p12q13)	4
		46,XX,inv(12)(p13q12)	1
		46,XX,inv(8)(p22;q21.3)	1
		46,XX,9phqh	1

Addition *n* = 1		*Male karyotypes*	
		46,XY,add(11)(q25)	1

Deletion *n* = 2		*Male karyotypes*	
		46,XY,del(8)(q12q22)/46,XY	1
		*Female karyotypes*	
		46,XX,del(18)(q21)	1

Chromosome marker *n* = 1		47,XY,+mar	1

Satellite chromosome *n* = 1		46,XY,22S+	1

**Table 4:** Chromosomal anomalies detected among 170 couples (*N* = number of couples) with reproductive disorders in Morocco.

Abnormalities	*N*	Females	Males	%	The most frequent chromosomal aberration (%)
Reciprocal translocations	6	2	4	3.5	—
Robertsonian translocations	2	2	0	1.2	—
Inversions	7	3	4	4.1	Inv(9)(p12;q13)(3,5%)
Aneuploidies	3	0	3	1.8	—
Total	18	7	11	10.6	

The percentage of abnormalities (%) is calculated in 170 couples.

**Table 5:** Comparison of frequencies of gonosomal abnormalities detected between the current study and other similar studies.

	Current study		[38]		[39]	
	Morocco		Oman		Morocco	
	*N* = 185		*N* = 298 *p* = 0.19		*N* = 1415 *p* ≤ 0.001	
	No. of patients	%	No. of patients	%	No. of patients	%
Klinefelter	47	25.4	73	24	20	1.41
Turner	57	30.8	112	38	133	9.39
Sex reversal	33	17.8	58	19.4	9	0.63

**Table 6:** Comparison of autosomal abnormalities detected in our report and other reports.

		F (%)	M (%)	Total (%)	References
Morocco *N* = 980	T of Abn	97	82	179	Current study
	Translocations	6 (6.19)	8 (9.76)	14 (7.82)	
	Inversions	8 (8.25)	5 (6.1)	13 (7.26)	
	Deletions	1 (1.03)	1 (1.21)	2 (1.11)	

Portugal *N* = 5978 *p* = 0.02	T of Abn	154	82	236	[28]
	Translocations	34 (22.07)	28 (34.14)	62 (26.27)	
	Inversions	8 (5.19)	7 (8.53)	15 (6.35)	
	Deletions	—	1 (1.21)	1 (0.42)	

Ukraine *N* = 3414 *p* = 0.06	T of Abn	34	47	81	[29]
	Translocations	22 (64.7)	27 (57.44)	49 (60.49)	
	Inversions	10 (29.41)	5 (10.63)	15 (18.51)	
	Deletions	—	—	—	

Egypt *N* = 5300 *p* ≤ 0.001	T of Abn	53	185	238	[21]
	Translocations	8 (15.09)	16 (8.64)	24 (10.08)	
	Inversions	10 (18.86)	4 (2.16)	14 (5.88)	
	Deletions	—	—	—	

T of Abn = total of abnormalities.

**Table 7:** Comparison of couple infertility in Morocco with other countries in the world.

Study	Country	Number of couples	Couples affected (%)	*p* value
Current study	Morocco	170	18 (10.6)	—
Elkarhat et al. [31]	Morocco	627	69 (11)	0.87
Pal et al. [41]	India	172	17 (9.88)	0.82
Ghazaey et al. [42]	Iran	728	43 (5.91)	0.02
Gonçalves et al. [43]	Brazil	151	11 (7.28)	0.30
Flynn et al. [44]	UK	795	28 (3.52)	≤0.001
Nazmy ([45]	Egypt	376	34 (9.04)	0.56
Elghezal et al. [46]	Tunis	1400	97 (6.92)	0.07

## Data Availability

The data that support the findings of this study are available from the corresponding author upon reasonable request.
